# Global Seroprevalence of Pre-existing Immunity Against AAV5 and Other AAV Serotypes in People with Hemophilia A

**DOI:** 10.1089/hum.2021.287

**Published:** 2022-04-19

**Authors:** Robert Klamroth, Gregory Hayes, Tatiana Andreeva, Keith Gregg, Takashi Suzuki, Ismail Haroon Mitha, Brandon Hardesty, Midori Shima, Toni Pollock, Patricia Slev, Johannes Oldenburg, Margareth C. Ozelo, Natalie Stieltjes, Sabine-Marie Castet, Johnny Mahlangu, Flora Peyvandi, Rashid Kazmi, Jean-François Schved, Andrew D. Leavitt, Michael Callaghan, Brigitte Pan-Petesch, Doris V. Quon, Jayson Andrews, Alex Trinh, Mingjin Li, Wing Yen Wong

**Affiliations:** ^1^ Comprehensive Care Haemophilia Treatment Center, Vivantes Klinikum im Friedrichshain, Berlin, Germany; ^2^ BioMarin Pharmaceutical, Inc., Novato, California, USA; ^3^ Municipal Center of Hemophilia Therapy, Saint Petersburg, Russia; ^4^ Ogikubo Hospital, Tokyo, Japan; ^5^ Worthwhile Clinical Trials, Lakeview Hospital, Benoni, South Africa; ^6^ Indiana Hemophilia and Thrombosis Center, Indianapolis, Indiana, USA; ^7^ Nara Medical University, Kashihara, Japan; ^8^ ARUP Laboratories, Salt Lake City, Utah, USA; ^9^ Universitätsklinikum Bonn, Bonn, Germany; ^10^ Hemocentro UNICAMP, Department of Internal Medicine, School of Medical Sciences, University of Campinas, Campinas, Brazil; ^11^ Department of Haematology and Regional Centre of Haemophilia Treatment, Hôpital Cochin, Assistance Publique Hôpitaux de Paris (AP-HP), Sorbonne Paris Cité, Université Paris Descartes, Paris, France; ^12^ Centre de Ressources et de Compétence des Maladies Hémorragiques Constitutionnelles, CHU de Bordeaux, Bordeaux, France; ^13^ Haemophilia Comprehensive Care Centre, University of the Witwatersrand and National Health Laboratory Service, Johannesburg, South Africa; ^14^ Fondazione IRCCS Ca’ Granda Ospedale Maggiore Policlinico, Angelo Bianchi Bonomi Hemophilia and Thrombosis Center, Milan, Italy; ^15^ Department of Pathophysiology and Transplantation, Università degli Studi di Milano, Milan, Italy; ^16^ Department of Haematology, Southampton University Hospital, Southampton, United Kingdom; ^17^ Centre Régional de Traitement des Hémophiles, Hôpital Saint-Eloi, CHRU de Montpellier, Montpellier, France; ^18^ Department of Medicine and Laboratory Medicine, University of California San Francisco, San Francisco, California, USA; ^19^ Division of Pediatric Hematology/Oncology, Central Michigan University, Detroit, Michigan, USA; ^20^ Centre Hospitalier Régional Universitaire de Brest, Hôpital A. Morvan, Brest, France; ^21^ Orthopaedic Hemophilia Treatment Center, Orthopaedic Institute for Children, Los Angeles, California, USA.

**Keywords:** gene therapy, hemophilia A, adeno-associated virus, antibody, seropositivity

## Abstract

Adeno-associated virus (AAV)-mediated gene therapy may provide durable protection from bleeding events and reduce treatment burden for people with hemophilia A (HA). However, pre-existing immunity against AAV may limit transduction efficiency and hence treatment success. Global data on the prevalence of AAV serotypes are limited. In this global, prospective, noninterventional study, we determined the prevalence of pre-existing immunity against AAV2, AAV5, AAV6, AAV8, and AAVrh10 among people ≥12 years of age with HA and residual FVIII levels ≤2 IU/dL. Antibodies against each serotype were detected using validated, electrochemiluminescent-based enzyme-linked immunosorbent assays. To evaluate changes in antibody titers over time, 20% of participants were retested at 3 and 6 months. In total, 546 participants with HA were enrolled at 19 sites in 9 countries. Mean (standard deviation) age at enrollment was 36.0 (14.87) years, including 12.5% younger than 18 years, and 20.0% 50 years of age and older. On day 1, global seroprevalence was 58.5% for AAV2, 34.8% for AAV5, 48.7% for AAV6, 45.6% for AAV8, and 46.0% for AAVrh10. Considerable geographic variability was observed in the prevalence of pre-existing antibodies against each serotype, but AAV5 consistently had the lowest seroprevalence across the countries studied. AAV5 seropositivity rates were 51.8% in South Africa (*n* = 56), 46.2% in Russia (*n* = 91), 40% in Italy (*n* = 20), 37.2% in France (*n* = 86), 26.8% in the United States (*n* = 71), 26.9% in Brazil (*n* = 26), 28.1% in Germany (*n* = 89), 29.8% in Japan (*n* = 84), and 5.9% in the United Kingdom (*n* = 17). For all serotypes, seropositivity tended to increase with age. Serostatus and antibody titer were generally stable over the 6-month sampling period. As clinical trials of AAV-mediated gene therapies progress, data on the natural prevalence of antibodies against various AAV serotypes may become increasingly important.

## Introduction

Hemophilia A (HA) is an X-linked bleeding disorder caused by mutations in the coagulation factor VIII (FVIII) gene.^1^ Severe HA (FVIII <1 IU/dL)^2^ is associated with bleeding in joints and soft tissues, leading to disabling arthropathy.^3^ Standard of care for severe HA—regular prophylaxis with exogenous FVIII—does not prevent breakthrough bleeding.^3,4^

As a monogenic disease, HA is a good candidate for gene therapy. Modest increases in FVIII levels ameliorate severe HA and provide clinically relevant improvements in disease phenotype.^5^ Because the functional levels of normal FVIII have a wide range, tightly regulated gene expression is not essential.^6–8^ Production of FVIII post-gene transduction can also be monitored with validated, quantitative assays.^9^

Adeno-associated viruses (AAV) are small, nonenveloped, single-stranded DNA viruses that are endemic and nonpathogenic in humans.^10^ Multiple AAV serotypes are known, each with their own tropism for particular cell types.^11^ AAVs are adapted for use as gene therapy vectors by removing the virus genome and replacing it with an expression cassette that includes tissue-selective regulatory elements and a gene of interest.^12^

In clinical trials, AAV-mediated gene therapy for HA such as valoctocogene roxaparvovec (AAV5-hFVIII-SQ) has provided durable protection from bleeding events for up to 5 years and has reduced treatment burden and increased quality of life.^13–15^ Clinical trials have screened for and, in some cases, limited eligibility to participants who do not have immunity against the AAV serotype of the vector being assessed.^14,16–18^ As AAV-mediated gene therapy for HA progresses toward regulatory approval and use outside of trials, pre-existing immunity to specific AAV serotypes may be a key factor that determines treatment eligibility. Even low levels of anti-AAV antibodies may reduce transduction of AAV vector gene therapy or heighten immune responses to the vector, leading to poor transduction of target cells and lack of therapeutic effect.^17,19–21^

Surveys of seroprevalence using diverse methods show pre-existing immunity against AAV varies geographically and by AAV serotype, both in the general population and in individuals with hemophilia.^22–25^ The broad range of available estimates reflects that most studies were conducted in a single country or center and assay methodology was not standardized. To estimate the global potential of AAV-mediated gene therapy, standardized assessment of AAV seroprevalence in both adults and adolescents with HA is needed. In this prospective global study, we determined the geographic distribution and titer stability of pre-existing immunity against AAV2, AAV5, AAV6, AAV8, and AAVrh10 capsids in adults and adolescents with HA in a central laboratory using comparable assays.

## Methods

### Participants

The study was conducted in people ≥12 years of age with HA and residual FVIII levels ≤2 IU/dL per medical history, who had previously been treated with FVIII concentrates. Participants could have been receiving treatment with prophylactic FVIII, on-demand FVIII, or both. People with and without FVIII inhibitors were eligible.

### Ethical study conduct

The protocol was approved by the institutional review boards or independent ethics committees of all participating sites. Participants provided informed consent before enrollment; minors provided assent and their parents/legally acceptable representatives provided informed consent before enrollment.

### Study design

In this noninterventional study, participants visited the clinic on day 1 for collection of biospecimens: plasma, serum, and peripheral blood mononuclear cells. Samples were processed at the local collection site, aliquoted, frozen at −70°C to −80°C, shipped to the biobank, and then sent to the central laboratory for analysis. Optional biospecimen retesting at 3 and/or 6 months was requested from ∼20% of participants across three titer ranges (negative, <100, and >100) in each country to evaluate antibody level consistency over time. No investigational product was administered as part of this study.

### Safety

Information about study procedure-related adverse reactions (ARs) was collected by contacting study participants (or their legally acceptable representatives) by telephone ∼24–72 h after biospecimen collection. The period for reporting ARs was from biospecimen collection until the time of the telephone call ∼24–72 h later.

### Total antibody assay methods

Measurement of antibodies against AAV5 was performed using a validated bridging total antibody (TAb) electrochemiluminescence (ECL) assay on the Meso Scale Discovery platform (MSD, Meso Scale Diagnostics, Rockville, MD; limit of detection [LOD], 33 ng/mL). An MSD plate was coated with unlabeled AAV5 capsid, plasma specimen was introduced, and ruthenylated (SULFO-TAG-labeled) capsid was added. If AAV5 antibodies are present in the plasma sample, they bind and thereby bridge the unlabeled and labeled capsids (Supplementary Fig. S1). After addition of the substrate tripropylamine and application of a voltage, an ECL signal is generated by positive samples.

Measurement of antibodies against the AAV2, AAV6, AAV8, and AAVrh10 serotypes was performed using qualified research-use-only (RUO) bridging ECL assays with similar design (LOD of 60, 15, 70, and 42 ng/mL, respectively). Recombinant capsids were generated at Virovek, Inc. (Hayward, CA). Assay validation or qualification and sample testing were performed at ARUP Laboratories (Salt Lake City, UT).

To determine sample positivity for AAV TAb, screening cut points targeting a 5% false positive rate were used. Cut points were statistically identified during assay validation/qualification. Response specificity was evaluated for positive samples by adding additional unlabeled capsids to compete with labeled capsids; positive samples were confirmed when the ECL assay signal inhibition from incubation with unlabeled capsids met or exceeded the pre-established confirmatory cut point.

Titers of confirmed positive samples were measured by serial dilution and interpolation at the titer cut point calculated during assay validation/qualification. Titer values are represented as the dilution at which the assay signal crossed the titer cut point. Positive samples with low titers that did not cross the cut point at the minimum required dilution of 20 were imputed as titer = 19.9 in subsequent analyses.

### Statistical methods

Sample size calculations were performed based on the expected rate of AAV5 seropositivity and the desired width of the confidence intervals (95% CI). Prevalence rate and corresponding CI were summarized. Prevalence rate by subgroup (*e.g.*, geographic region or other baseline factors) and prevalence rate over time for the retesting subgroup were also evaluated. Global HA weighted average was calculated by multiplying the percentage of participants who tested positive in each country by the number of people with HA in that country per 2018 World Federation of Hemophilia (WFH) survey divided by the total number of people with HA in all the countries in this study per WFH survey.^26^

## Results

### Study participants

In total, 546 participants with HA, including 478 adults (≥18 years of age) and 68 adolescents (<18 years of age), were enrolled at 19 sites in 9 countries; 542 participants completed the study (99.3%), 3 were lost to follow-up, and 1 discontinued by physician decision. Mean (standard deviation [SD]) age at enrollment was 36.0 (14.87) years, including 12.5% younger than 18 years and 6.2% at 60 years of age and older (Table 1); 23.3% of participants were between >30 and ≤40 years of age, while 26.9% were between ≥18 and ≤30 years of age. Mean age at enrollment varied from 28.4 years in Brazil to 48.7 years in Italy (Supplementary Table S1). At baseline, more participants were receiving prophylactic FVIII (80.0%) than on-demand FVIII (20.0%).

**Table 1.** Participant demographics and baseline characteristics

Parameter	Overall Population (*N* = 546)
Age at enrollment, mean ± SD, years	36.0 ± 14.9
Age at enrollment, *n* (%)	
12 to <18 years	68 (12.5)
≥18 to ≤30 years	147 (26.9)
>30 to ≤40 years	127 (23.3)
>40 to ≤50 years	103 (18.9)
>50 to ≤60 years	67 (12.3)
>60 years	34 (6.2)
Sex, *n* (%)	
Male	542 (99.3)
Female	4 (0.7)
Race, *n* (%)	
Asian	91 (16.7)
Black or African American	66 (12.1)
White	293 (53.7)
Native Hawaiian or other Pacific Islander	1 (0.2)
Not provided due to patient privacy rules	95 (17.4)
Ethnicity, *n* (%)	
Hispanic or Latino	14 (2.6)
Not Hispanic or Latino	501 (91.8)
Missing	31 (5.7)
Time since hemophilia diagnosis, mean ± SD, years	31.4 ± 14.7
History of exposure to hepatitis B, *n* (%)	103 (18.9)
History of exposure to hepatitis C, *n* (%)	282 (51.6)
Type of FVIII treatment, *n* (%)	
On demand	109 (20.0)
Prophylaxis	437 (80.0)
Baseline FVIII activity, mean ± SD, IU/dL	0.7 ± 0.6
Medical history conditions, *n* (%)^a^	
Hemophilic arthropathy	199 (36.4)
Hypertension	67 (12.3)
HIV infection	62 (11.4)
Arthropathy	45 (8.2)
Knee arthroplasty	42 (7.7)
Synovectomy	27 (4.9)
Synoviorthesis	21 (3.8)
Central venous catheterization	20 (3.7)
Drug hypersensitivity	20 (3.7)
Hip arthroplasty	19 (3.5)
Chronic hepatitis C	19 (3.5)

^a^ Reported in ≥3% of participants.

FVIII, factor VIII; SD, standard deviation.

### Overall AAV seropositivity

**Figure 1.**
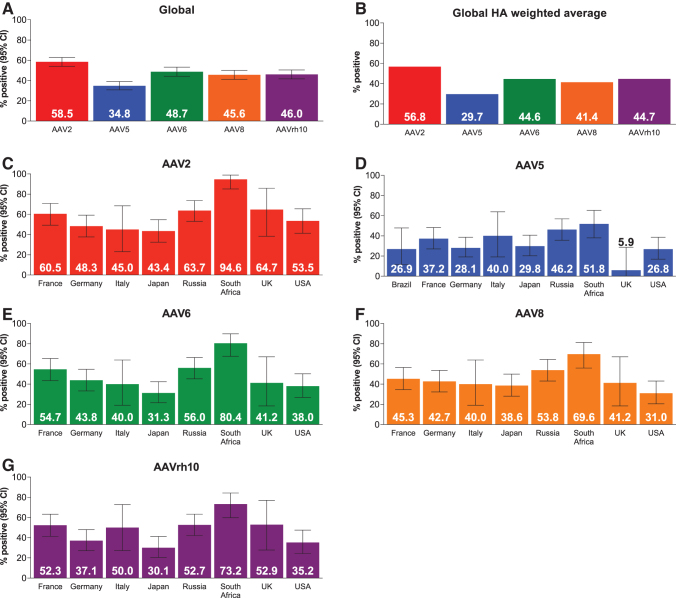
Seropositivity for **(A)** the global population, **(B)** using the global HA weighted average, and by country for **(C)** AAV2, **(D)** AAV5, **(E)** AAV6, **(F)** AAV8, and **(G)** AAVrh10. Data are for adults and adolescents on day 1. Samples from Brazil were only tested using the AAV5 assay, not RUO assays. Global HA weighted average was calculated by multiplying the percentage of participants who tested positive in each country by the number of people with HA in that country, per 2018 WFH survey, divided by the total number of people with HA in all countries in this study, per 2018 WFH survey. AAV, adeno-associated virus; CI, confidence interval; HA, hemophilia A; RUO, research-use-only; WFH, World Federation of Hemophilia.

Among the 540 participants with nonmissing AAV5 TAb assessments on day 1, 34.8% were positive for anti-AAV5 antibodies (Fig. 1 and Supplementary Table S2). Factoring in the prevalence of HA in the countries being assayed, the global weighted average of AAV5 seroprevalence in people with HA was 29.7%. For other AAV serotypes, global seroprevalence was 58.5% for AAV2, 48.7% for AAV6, 45.6% for AAV8, and 46.0% for AAVrh10. Global HA weighted average was 56.8% for AAV2, 44.6% for AAV6, 41.4% for AAV8, and 44.7% for AAVrh10.

### Geographic variability

There was considerable geographic variability in the prevalence of pre-existing antibodies against AAV5 (Fig. 1 and Supplementary Table S2). Countries with seropositivity rates 30% or less, roughly the global weighted average, included the United Kingdom (5.9%, *n* = 17), the United States (26.8%, *n* = 71), Brazil (26.9%, *n* = 26), Germany (28.1%, *n* = 89), and Japan (29.8%, *n* = 84). Countries with seropositivity rates above 30% included South Africa (51.8%, *n* = 56), Russia (46.2%, *n* = 91), Italy (40%, *n* = 20), and France (37.2%, *n* = 86).

There was geographic variation in seropositivity for other serotypes, which were all at a higher prevalence than for AAV5. Countries with AAV2 seropositivity rates over 60% included France (60.5%), Russia (63.7%), South Africa (94.6%), and the United Kingdom (64.7%). Seropositivity rates for AAV6, AAV8, and AAVrh10 were also over 60% in South Africa. Seropositivity to AAV2, AAV6, AAV8, and AAVrh10 was not assessed in Brazil due to the exploratory nature of the analyses and the regional requirement that study results be disclosed to participants.

### Seropositivity by age

**Figure 2.**
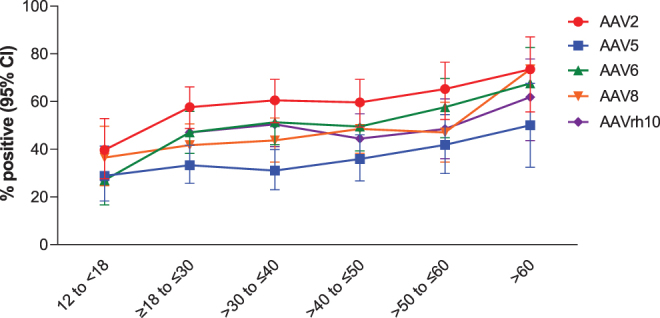
AAV serotype positivity by age group. Data are for the global population on day 1.

Globally, 35.7% of adult participants were positive for AAV5 antibodies, compared to 28.8% of adolescent participants (Supplementary Table S2). Seropositivity rates were higher in adults versus adolescents for other serotypes as well. For all serotypes, seropositivity tended to increase with age (Fig. 2).

### Antibody titers

**Figure 3.**
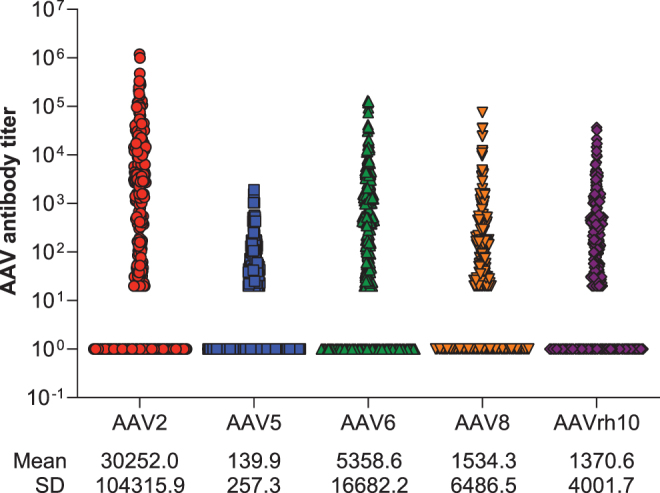
Individual participant titers on day 1. Data are dilution titers from day 1 for individual participants in the global population. Participants with negative titers are shown as 1. Participants with positive titer results and a titer <20, the MRD, are shown as 20. Width is representative of the number of points at a particular value. Mean (SD) values are for participants with quantifiable titers only: AAV2, *n* = 294; AAV5, *n* = 188; AAV6, *n* = 247; AAV8, *n* = 227; AAVrh10, *n* = 233. MRD, minimum required dilution; SD, standard deviation.

Mean (SD) AAV5 TAb titer, as measured by serial dilution, was 139.9 (257.34) on day 1; median (range) titer on day 1 was 53.5 (19.9, 1913.0) (Fig. 3). Mean titers for the other serotypes were at least one order of magnitude higher. For seropositive individuals, titer differed across geographic regions (Supplementary Fig. S2). Mean (SD) titer of AAV5 seropositive participants was lowest in the United Kingdom, the United States, Germany, and Brazil, while it was highest in South Africa, Russia, Italy, and France.

### Stability of seropositivity

**Figure 4.**
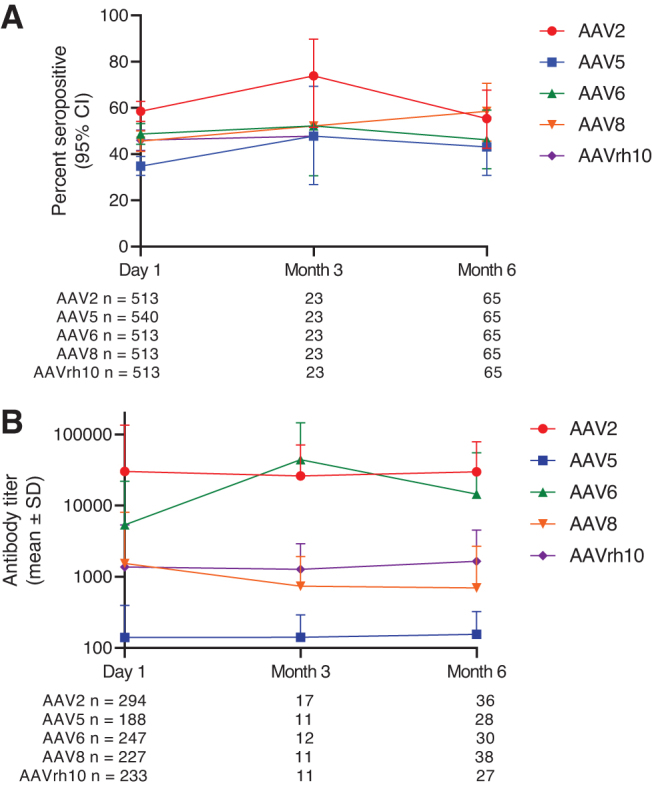
For all serotypes over 6 months, **(A)** percent of positive participants and **(B)** mean antibody titers in positive participants. Data are for all participants in the global population with valid assay results for the relevant time point. Titer was only evaluated in seropositive participants. Note that titers cannot be directly compared across serotypes, as assays have varying sensitivity.

Of the 72 participants in the global population, who were also assessed at time points after day 1, 49 were assessed at month 6 only, 7 were assessed at month 3 only, and 16 were assessed at month 3 and 6. Over the 6-month sampling period, serostatus was generally stable for all serotypes (Fig. 4A), but some participants shifted from negative to positive or vice versa.

For AAV5 TAb, 2 and 3 participants shifted from negative on day 1 to positive at month 3 and 6, respectively (2 in Germany, 2 in Japan, and 1 in Russia). These participants had titer values under 150 at visits where they tested positive (7 titers were <20, 1 was 62, 1 was 149, and 1 had values of 21 and 25 at different time points). Seroconversion from negative to positive occurred during the study period in 3 participants for antibodies to AAV2 (1 each in Russia, South Africa, and the United States), 1 participant in Japan for antibodies to AAV6, 11 participants for antibodies to AAV8 (7 in Germany, 2 in Japan, 1 in Russia, and 1 in the United States), and 0 participants for antibodies to AAVrh10.

For AAV5 TAb, 2 and 3 participants shifted from positive on day 1 to negative at month 3 and 6, respectively (1 in Germany, 1 in Japan, 2 in Russia, and 1 in South Africa). A shift from positive to negative seropositivity occurred during the study period in 6 participants for AAV2 antibodies (1 in France, 3 in Germany, 1 in Russia, and 1 in the United States), 6 participants for AAV6 antibodies (2 in France and 4 in Russia), 1 participant in South Africa for AAV8 antibodies, and 2 participants for AAVrh10 antibodies (1 each in Germany and Russia).

Antibody titer was generally stable over time for all serotypes (Fig. 4B). For AAV5, antibody titer on day 1 was well correlated with titer at 3 months (*R*^2^ = 0.808, *p* < 0.0001) and 6 months (*R*^2^ = 0.592, *p* < 0.0001). While direct comparisons between titers across serotypes should be performed with caution as assays have varying sensitivity, AAV5 antibody titers among seropositive participants were 1–2 orders of magnitude lower compared to other serotypes.

### Immunity against multiple serotypes

The number of individual participants seropositive for multiple serotypes on day 1 was analyzed (Table 2). Of 513 participants with nonmissing results for all serotypes, 125 participants (24.4%) were positive for antibodies to all serotypes. Similarly, 126 participants (24.6%) were negative for antibodies to all serotypes. A total of 60 (11.7%) participants were negative for AAV5 TAb, but positive for TAb to all 4 other serotypes, while 22 (4.3%) participants were positive for AAV5 TAb, but negative for all others. The remaining 180 participants (35.1%) were seropositive for 24 various combinations of serotypes.

**Table 2.** Cross-tabulation of adeno-associated virus seropositivity on day 1 in decreasing order of frequency

AAV5	AAV2	AAV6	AAV8	AAVrh10	*n* (%)	Cumulative,* n *(%)
−	−	−	−	−	126 (24.6)	126 (24.6)
+	+	+	+	+	125 (24.4)	251 (48.9)
−	+	+	+	+	60 (11.7)	311 (60.6)
−	+	−	−	−	51 (9.9)	362 (70.6)
+	−	−	−	−	22 (4.3)	384 (74.9)
−	−	−	+	−	18 (3.5)	402 (78.4)
−	+	+	−	−	14 (2.7)	416 (81.1)
−	−	−	−	+	13 (2.5)	429 (83.6)
−	+	+	−	+	11 (2.1)	440 (85.8)
−	−	+	−	−	11 (2.1)	451 (87.9)
+	+	−	−	−	9 (1.8)	460 (89.7)
+	+	+	−	+	8 (1.6)	468 (91.2)
−	−	−	+	+	6 (1.2)	474 (92.4)
−	+	+	+	−	5 (1.0)	479 (93.4)
−	+	−	−	+	5 (1.0)	484 (94.3)
+	−	−	+	−	4 (0.8)	488 (95.1)
−	+	−	+	−	4 (0.8)	492 (95.9)
+	+	+	−	−	3 (0.6)	495 (96.5)
+	−	+	+	−	3 (0.6)	498 (97.1)
−	−	+	+	−	3 (0.6)	501 (97.7)
+	+	+	+	−	2 (0.4)	503 (98.1)
+	−	+	−	−	2 (0.4)	505 (98.4)
−	+	−	+	+	2 (0.4)	507 (98.8)
−	−	+	−	+	2 (0.4)	509 (99.2)
+	+	−	−	+	1 (0.2)	510 (99.4)
+	−	−	+	+	1 (0.2)	511 (99.6)
+	−	−	−	+	1 (0.2)	512 (99.8)
−	−	+	+	+	1 (0.2)	513 (100.0)

This analysis includes 513 participants with nonmissing results for all serotypes.

AAV, adeno-associated virus.

### Seropositivity by infection history

When seropositivity on day 1 was analyzed by history of HIV, hepatitis B, and hepatitis C infection, seropositivity rates for both positive and negative participants were similar to the overall global population (Supplementary Table S3). HIV-positive participants had lower AAV5, AAV6, and AAVrh10 seropositivity rates than HIV-negative participants, and participants with history of hepatitis B had lower AAV2, AAV6, and AAVrh10 seropositivity rates than participants without such history. Participants with history of hepatitis C had slightly higher seropositivity rates for all serotypes than those without history of hepatitis C.

### Safety

Four ARs were reported by 4 participants (0.7%); all were Grade 1 and resolved without sequelae between 4 and 8 days after onset. The 4 ARs included 2 events of subcutaneous hemorrhage and 1 event each of erythema and bruising (contusion). No serious AR was reported.

## Discussion

To our knowledge, this is the largest study to date on the seroprevalence of AAV antibodies in people with HA. AAV-mediated gene therapy has the potential to transform treatment of HA by reducing the frequency of bleeding, reducing treatment burden, FVIII utilization, and infusion frequency, and increasing quality of life. Pre-existing immunity to the AAV serotype of a gene therapy vector may lead to poor transduction of target cells and lack of therapeutic effect, and therefore limit treatment eligibility.^17,19–21^ We found that pre-existing AAV immunity varied across serotypes and countries, but global seropositivity was lowest for AAV5 and highest for AAV2. These findings have implications for the global potential of AAV gene therapy for HA.

AAV antibody assays are not standardized, and thresholds for positivity are assay specific. Results from TAb assays have been shown to successfully predict AAV5 transduction in nonhuman primates.^19,27^ TAb assays are considered less variable/more precise than cell-based neutralization (NAb) or transduction inhibition (TI) assays, although analyses of 100 plasma samples found 71% concordance between the 2 methods.^28^ Some AAV-based gene therapy programs have used cell-based NAb or TI assays to measure seropositivity at baseline, but have dosed participants irrespective of pre-existing immunity.^29^ The relevance of the NAb and TI assay results to transduction and transgene expression should become clear as more data from these programs are published.

The geographic variability in seropositivity observed in this study is mostly in line with results from previous studies, both in the general population and individuals with hemophilia.^23,30–32^ The estimated seroprevalence of anti-AAV5 immunity in the United Kingdom observed in this study is lower than previously reported. In a larger sample (*n* = 100) of HA participants drawn from 7 United Kingdom hemophilia centers, 21% were AAV5 TAb positive, with titers ranging from 57 to 2,246.^25^ Unlike previous studies focused on a single geography or serotype, in this study, we collected data across multiple countries using the same assays at a central laboratory. These standardized data from a prospective, global study allow more robust direct comparisons of seropositivity rates across geographic regions than were available previously.

In this study, seropositivity rates were higher in adult versus adolescent participants, and seropositivity was also higher in older adults than in younger adults. These results are similar to previous observations.^24^ Given that AAV is an endemic virus, a higher rate of exposure in older individuals is to be expected. Over the 6-month course of the study, very few participants seroconverted, and no geographic trend was apparent. Higher age of the participants in some geographies such as Italy and Japan may have influenced results. There was a trend suggesting participants with history of hepatitis C may have higher rates of seropositivity than those with no history of hepatitis C.

In this study, about a quarter of participants were positive for all AAV serotypes assessed; a substantial proportion of people with HA may therefore have difficulty accessing and benefiting from gene therapy using any of the most commonly used AAV vectors.^33^ Administration of AAV5-based gene therapy produced an increase in cross-reactive antibodies to AAV2, AAV6, AAV8, and AAVrh10.^33^ Titers of these cross-reactive antibodies were several orders of magnitude lower than corresponding anti-AAV5 TAb titers, and they declined over time.^33^ Mitigation strategies could potentially be used to facilitate dosing of seropositive patients, particularly those with low antibody titers, but such approaches are currently experimental.^8,34^

More research is necessary to characterize immune factors that may inhibit transduction and determine the threshold at which anti-AAV antibodies begin to affect the efficacy of gene therapy using AAV vectors. A phase 1/2 trial of valoctocogene roxaparvovec, an AAV5 gene therapy for HA, is underway in AAV5-seropositive participants (NCT03520712) and will provide further information on efficacy of gene therapy in seropositive individuals.

Limitations of this analysis include small sample sizes within subanalysis groups and partial sampling of participants at later time points. Additional research in larger samples is needed on the dynamics of seropositivity over time. We did not assess seasonality of AAV infection. In addition, we lack data on time points >6 months, and further study of the persistence of anti-AAV antibody responses is warranted.

We only examined humoral immunity against AAV serotypes; we did not examine whether participants carried AAV-specific cellular immune responses. Wild-type AAV is a weak immunogen that generally stimulates only transient cellular responses with poor memory following natural infection. These cellular responses have been difficult to detect in peripheral blood, usually requiring several rounds of *in vitro* expansion with recombinant AAV peptide libraries.^35^

As AAV-mediated gene therapies for HA continue along the development pathway, data on pre-existing immunity against AAV serotypes will prove invaluable for informing ongoing development and determining who can benefit from such treatments once they are available. The results of this study support the value of AAV5 as a gene therapy vector. AAV5 not only shows a lower global prevalence of pre-existing immunity compared to other serotypes but it also has a maximum Ab titer that is 1–2 orders of magnitude lower than AAV2 or AAV8.

## Supplementary Material

Supplemental data

Supplemental data

Supplemental data

Supplemental data

Supplemental data

## Data Availability

De-identified individual participant data underlying these results will be made available, together with the clinical protocol and data dictionaries, for noncommercial, academic purposes. Additional supporting documents may be available upon request. Investigators will be able to request access to these data and supporting documents through the Publication Data Request page at www.BioMarin.com beginning 6 months and ending 2 years after publication. Data associated with any ongoing development program will be made available within 6 months after approval of the relevant product. Requests must include a research proposal clarifying how the data will be used, including proposed analysis methodology. Research proposals will be evaluated relative to publicly available criteria available at www.BioMarin.com to determine if access will be given, contingent upon execution of a data access agreement with BioMarin Pharmaceutical, Inc.

## References

[1] Iorio A, Stonebraker JS, Chambost H, et al. Establishing the prevalence and prevalence at birth of hemophilia in males: a meta-analytic approach using national registries. Ann Intern Med 2019;171:540–546. 31499529 10.7326/M19-1208

[2] Blanchette VS, Key NS, Ljung LR, et al. Definitions in hemophilia: communication from the SSC of the ISTH. J Thromb Haemost 2014;12:1935–1939. 25059285 10.1111/jth.12672

[3] Srivastava A, Santagostino E, Dougall A, et al. WFH guidelines for the management of hemophilia, 3rd edition. Haemophilia 2020;26 Suppl 6:1–158. 32744769 10.1111/hae.14046

[4] Berntorp E, Dolan G, Hay C, et al. European retrospective study of real-life haemophilia treatment. Haemophilia 2017;23:105–114. 27761962 10.1111/hae.13111

[5] Mannucci PM, Tuddenham EG. The hemophilias—from royal genes to gene therapy. N Engl J Med 2001;344:1773–1779. 11396445 10.1056/NEJM200106073442307

[6] Roth DA, Tawa NE Jr., O'Brien JM, et al. Nonviral transfer of the gene encoding coagulation factor VIII in patients with severe hemophilia A. N Engl J Med 2001;344:1735–1742. 11396439 10.1056/NEJM200106073442301

[7] Batty P, Lillicrap D. Advances and challenges for hemophilia gene therapy. Hum Mol Genet 2019;28:R95–R101. 31332444 10.1093/hmg/ddz157

[8] Doshi BS, Arruda VR. Gene therapy for hemophilia: what does the future hold? Ther Adv Hematol 2018;9:273–293. 30210756 10.1177/2040620718791933 PMC6130099

[9] Peyvandi F, Oldenburg J, Friedman KD. A critical appraisal of one-stage and chromogenic assays of factor VIII activity. J Thromb Haemost 2016;14:248–261. 26663865 10.1111/jth.13215

[10] Colella P, Ronzitti G, Mingozzi F. Emerging issues in AAV-mediated in vivo gene therapy. Mol Ther Methods Clin Dev 2018;8:87–104. 29326962 10.1016/j.omtm.2017.11.007 PMC5758940

[11] Daya S, Berns KI. Gene therapy using adeno-associated virus vectors. Clin Microbiol Rev 2008;21:583–593. 18854481 10.1128/CMR.00008-08 PMC2570152

[12] Wang D, Tai PWL, Gao G. Adeno-associated virus vector as a platform for gene therapy delivery. Nat Rev Drug Discov 2019;18:358–378. 30710128 10.1038/s41573-019-0012-9 PMC6927556

[13] Pasi KJ, Rangarajan S, Mitchell N, et al. Multiyear follow-up of AAV5-hFVIII-SQ gene therapy for hemophilia A. N Engl J Med 2020;382:29–40. 31893514 10.1056/NEJMoa1908490

[14] Rangarajan S, Walsh L, Lester W, et al. AAV5-factor VIII gene transfer in severe hemophilia A. N Engl J Med 2017;377:2519–2530. 29224506 10.1056/NEJMoa1708483

[15] Ozelo MC, Mahlangu J, Pasi KJ, et al. Efficacy and safety of valoctocogene roxaparvovec adeno-associated virus gene transfer for severe hemophilia A: results from the phase 3 GENEr8-1 trial. Res Pract Thromb Haemost 2021;5:e12591.

[16] Nathwani AC, Tuddenham EG, Rangarajan S, et al. Adenovirus-associated virus vector-mediated gene transfer in hemophilia B. N Engl J Med 2011;365:2357–2365. 22149959 10.1056/NEJMoa1108046 PMC3265081

[17] George LA, Sullivan SK, Giermasz A, et al. Hemophilia B gene therapy with a high-specific-activity factor IX variant. N Engl J Med 2017;377:2215–2227. 29211678 10.1056/NEJMoa1708538 PMC6029626

[18] Miesbach W, Meijer K, Coppens M, et al. Gene therapy with adeno-associated virus vector 5-human factor IX in adults with hemophilia B. Blood 2018;131:1022–1031. 29246900 10.1182/blood-2017-09-804419 PMC5833265

[19] Long BR, Sandza K, Holcomb J, et al. The impact of pre-existing immunity on the non-clinical pharmacodynamics of AAV5-based gene therapy. Mol Ther Methods Clin Dev 2019;13:440–452. 31193016 10.1016/j.omtm.2019.03.006 PMC6513774

[20] Manno CS, Pierce GF, Arruda VR, et al. Successful transduction of liver in hemophilia by AAV-Factor IX and limitations imposed by the host immune response. Nat Med 2006;12:342–347. 16474400 10.1038/nm1358

[21] Gao G, Lu Y, Calcedo R, et al. Biology of AAV serotype vectors in liver-directed gene transfer to nonhuman primates. Mol Ther 2006;13:77–87. 16219492 10.1016/j.ymthe.2005.08.017

[22] Boutin S, Monteilhet V, Veron P, et al. Prevalence of serum IgG and neutralizing factors against adeno-associated virus (AAV) types 1, 2, 5, 6, 8, and 9 in the healthy population: implications for gene therapy using AAV vectors. Hum Gene Ther 2010;21:704–712. 20095819 10.1089/hum.2009.182

[23] Calcedo R, Vandenberghe LH, Gao G, et al. Worldwide epidemiology of neutralizing antibodies to adeno-associated viruses. J Infect Dis 2009;199:381–390. 19133809 10.1086/595830 PMC10826927

[24] Li C, Narkbunnam N, Samulski RJ, et al. Neutralizing antibodies against adeno-associated virus examined prospectively in pediatric patients with hemophilia. Gene Ther 2012;19:288–294. 21697954 10.1038/gt.2011.90

[25] Stanford S, Pink R, Creagh D, et al. Adenovirus-associated antibodies in UK cohort of hemophilia patients: a seroprevalence study of the presence of adenovirus-associated virus vector-serotypes AAV5 and AAV8 neutralizing activity and antibodies in patients with hemophilia A. Res Pract Thromb Haemost 2019;3:261–267. 31011710 10.1002/rth2.12177 PMC6462753

[26] World Federation of Hemophilia. Report on the Annual Global Survey 2018. 2019. www1.wfh.org/publications/files/pdf-1731.pdf (last accessed September 15, 2021).

[27] Gorovits B, Azadeh M, Buchlis G, et al. Evaluation of the humoral response to adeno-associated virus-based gene therapy modalities using total antibody assays. AAPS J 2021;23:108. 34529177 10.1208/s12248-021-00628-3 PMC8445016

[28] Falese L, Sandza K, Yates B, et al. Strategy to detect pre-existing immunity to AAV gene therapy. Gene Ther 2017;24:768–778. 29106404 10.1038/gt.2017.95 PMC5746592

[29] Recht M, Leebeek FWG, Miesbach W, et al. Clinical outcomes in patients with and without pre-existing neutralizing antibodies to the vector: 6month data from the phase 3 HOPE-B gene therapy trial of etranacogene dezaparvovec. Mol Ther 2021;29:Abstract 88.

[30] Kruzik A, Fetahagic D, Hartlieb B, et al. Prevalence of anti-adeno-associated virus immune responses in international cohorts of healthy donors. Mol Ther Methods Clin Dev 2019;14:126–133. 31338384 10.1016/j.omtm.2019.05.014 PMC6629972

[31] Liu Q, Huang W, Zhang H, et al. Neutralizing antibodies against AAV2, AAV5 and AAV8 in healthy and HIV-1-infected subjects in China: implications for gene therapy using AAV vectors. Gene Ther 2014;21:732–738. 24849042 10.1038/gt.2014.47

[32] Mimuro J, Mizukami H, Shima M, et al. The prevalence of neutralizing antibodies against adeno-associated virus capsids is reduced in young Japanese individuals. J Med Virol 2014;86:1990–1997. 24136735 10.1002/jmv.23818

[33] Long BR, Veron P, Kuranda K, et al. Early phase clinical immunogenicity of valoctocogene roxaparvovec, an AAV5-mediated gene therapy for hemophilia A. Mol Ther 2021;29:597–610. 33309883 10.1016/j.ymthe.2020.12.008 PMC7854299

[34] Verdera HC, Kuranda K, Mingozzi F. AAV Vector immunogenicity in humans: a long journey to successful gene transfer. Mol Ther 2020;28:723–746. 31972133 10.1016/j.ymthe.2019.12.010 PMC7054726

[35] Ronzitti G, Gross DA, Mingozzi F. Human immune responses to adeno-associated virus (AAV) vectors. Front Immunol 2020;11:670. 32362898 10.3389/fimmu.2020.00670 PMC7181373

