# Supplementary material for: Global Seroprevalence of Pre-existing Immunity Against AAV5 and Other AAV Serotypes in People with Hemophilia A
Source: Hum Gene Ther. 2022 Apr 19;33(7-8):432–41. doi: 10.1089/hum.2021.287 (PMC9063149; doi:10.1089/hum.2021.287)
Supplement: Supplemental data [file Suppl_FigS1.docx]

**Supplementary Figure 1.** Schematic of the validated bridging TAb ECL assay used to identify positive samples


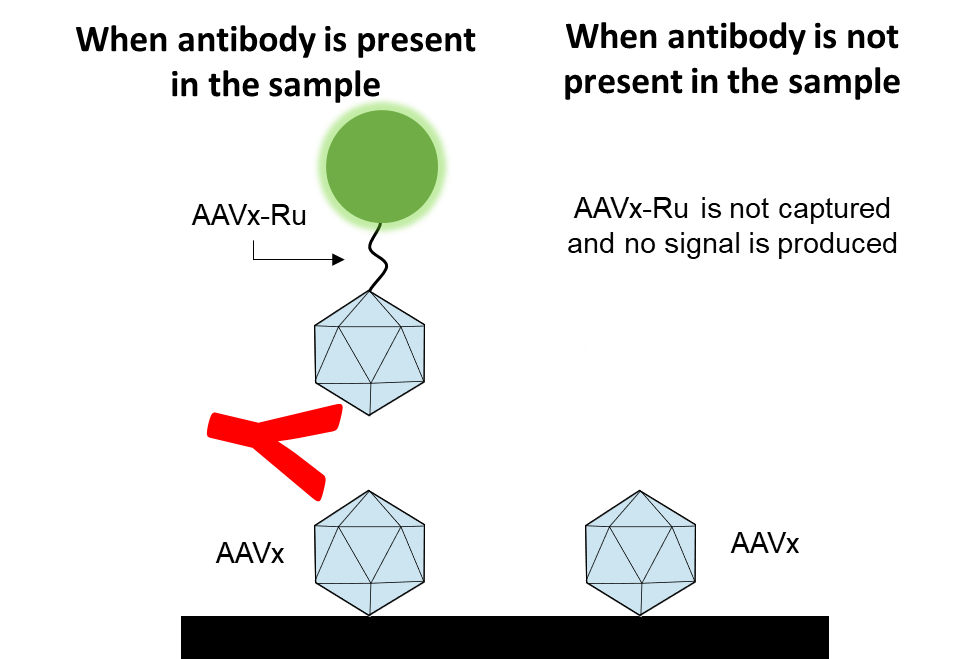


AAVx, adeno-associated virus serotype X; AAVx-Ru, ruthenylated AAVx capsid; ECL, electrochemiluminescence; TAb, total antibody.
