# Supplementary material for: Global Seroprevalence of Pre-existing Immunity Against AAV5 and Other AAV Serotypes in People with Hemophilia A
Source: Hum Gene Ther. 2022 Apr 19;33(7-8):432–41. doi: 10.1089/hum.2021.287 (PMC9063149; doi:10.1089/hum.2021.287)
Supplement: Supplemental data [file Suppl_TableS1.docx]

# SUPPLEMENTARY MATERIAL

**Supplementary Table 1.** Participant demographics and clinical characteristics by country

| **Parameter** | **Brazil**  **(n = 26)** | **France**  **(n = 87)** | **Germany**  **(n = 90)** | **Italy**  **(n = 20)** | **Japan**  **(n = 84)** | **Russia**  **(n = 91)** | **South Africa**  **(n = 60)** | **UK**  **(n = 17)** | **US**  **(n = 71)** |
| --- | --- | --- | --- | --- | --- | --- | --- | --- | --- |
| Age at enrollment, mean ± SD years | 28.4 ± 10.9 | 37.0 ± 15.1 | 38.0 ± 16.2 | 48.7 ± 12.8 | 40.9 ± 15.0 | 32.9 ± 13.1 | 32.9 ± 13.8 | 37.0 ± 16.6 | 32.0 ± 13.1 |
| Age at enrollment, n (%) |  |  |  |  |  |  |  |  |  |
| 12 to <18 years | 3 (11.5) | 12 (13.8) | 14 (15.6) | 0 | 8 (9.5) | 16 (17.6) | 5 (8.3) | 1 (5.9) | 9 (12.7) |
| ≥18 to ≤30 years | 12 (46.2) | 19 (21.8) | 13 (14.4) | 1 (5.0) | 15 (17.9) | 24 (26.4) | 30 (50.0) | 5 (29.4) | 28 (39.4) |
| >30 to ≤40 years | 6 (23.1) | 20 (23.0) | 22 (24.4) | 4 (20.0) | 16 (19.0) | 27 (29.7) | 10 (16.7) | 5 (29.4) | 17 (23.9) |
| >40 to ≤50 years | 4 (15.4) | 18 (20.7) | 20 (22.2) | 6 (30.0) | 24 (28.6) | 14 (15.4) | 7 (11.7) | 2 (11.8) | 8 (11.3) |
| >50 to ≤60 years | 1 (3.8) | 13 (14.9) | 13 (14.4) | 4 (20.0) | 12 (14.3) | 10 (11.0) | 4 (6.7) | 3 (17.6) | 7 (9.9) |
| >60 years | 0 | 5 (5.7) | 8 (8.9) | 5 (25.0) | 9 (10.7) | 0 | 4 (6.7) | 1 (5.9) | 2 (2.8) |
| Sex, male, n (%) | 26 (100) | 87 (100) | 89 (98.9) | 20 (100) | 84 (100) | 91 (100) | 59 (98.3) | 17 (100) | 69 (97.2) |
| Race, n (%) |  |  |  |  |  |  |  |  |  |
| Asian | 0 | 0 | 0 | 0 | 84 (100) | 0 | 1 (1.7) | 2 (11.8) | 4 (5.6) |
| Black or African American | 5 (19.2) | 0 | 0 | 0 | 0 | 0 | 53 (88.3) | 0 | 8 (11.3) |
| White | 21 (80.8) | 9 (10.3) | 90 (100) | 20 (100) | 0 | 90 (98.9) | 6 (10.0) | 15 (88.2) | 42 (59.2) |
| Native Hawaiian or other Pacific Islander | 0 | 0 | 0 | 0 | 0 | 1 (1.1) | 0 | 0 | 0 |
| Not provided due to patient privacy rules | 0 | 78 (89.7) | 0 | 0 | 0 | 0 | 0 | 0 | 17 (23.9) |
| Hispanic or Latino ethnicity, n (%) | 5 (19.2) | 0 | 2 (2.2) | 0 | 1 (1.2) | 0 | 0 | 17 (100) | 6 (8.5) |
| History of exposure to hepatitis B, n (%) | 2 (7.7) | 24 (27.6) | 22 (24.4) | 13 (65.0) | 28 (33.3) | 2 (2.2) | 0 | 4 (23.5) | 8 (11.3) |
| History of exposure to hepatitis C, n (%) | 7 (26.9) | 48 (55.2) | 50 (55.6) | 19 (95.0) | 55 (65.5) | 58 (63.7) | 5 (8.3) | 8 (47.1) | 32 (45.1) |
| Time since hemophilia diagnosis, mean ± SD years | 26.1 ± 9.0 | 34.6 ± 14.7 | 35.7 ± 15.3 | 41.4 ± 8.3 | 34.5 ± 15.1 | 30.7 ± 12.4 | 18.1 ± 13.6 | 36.5 ± 16.2 | 29.8 ± 13.0 |
| Type of FVIII treatment, n (%) |  |  |  |  |  |  |  |  |  |
| On-demand | 0 | 22 (25.3) | 6 (6.7) | 5 (25.0) | 5 (6.0) | 21 (23.1) | 40 (66.7) | 3 (17.7) | 7 (9.9) |
| Prophylaxis | 26 (100) | 65 (74.7) | 84 (93.3) | 5 (75.0) | 79 (94.0) | 70 (76.9) | 20 (33.3) | 14 (82.3) | 84 (90.1) |

FVIII, factor VIII; SD, standard deviation.
