# Supplementary material for: Global Seroprevalence of Pre-existing Immunity Against AAV5 and Other AAV Serotypes in People with Hemophilia A
Source: Hum Gene Ther. 2022 Apr 19;33(7-8):432–41. doi: 10.1089/hum.2021.287 (PMC9063149; doi:10.1089/hum.2021.287)
Supplement: Supplemental data [file Suppl_TableS2.docx]

**Supplementary Table 2.** AAV serotype Day 1 positivity frequency overall and by country and age

| **AAV Serotype** | **Global** | **Brazil** | **France** | **Germany** | **Italy** | **Japan** | **Russia** | **South Africa** | **UK** | **US** |
| --- | --- | --- | --- | --- | --- | --- | --- | --- | --- | --- |
| **AAV2 %**  **(+/total)** | 58.5 (300/513) | N/A | 60.5  (52/86) | 48.3  (43/89) | 45.0  (9/20) | 43.4  (36/83) | 63.7  (58/91) | 94.6  (53/56) | 64.7  (11/17) | 53.5  (38/71) |
| Adolescents | 39.7 (25/63) | N/A | 36.4  (4/11) | 7.1  (1/14) | 0/0 | 50.0  (4/8) | 37.5  (6/16) | 100  (4/4) | 0/1 | 66.7  (6/9) |
| Adults | 61.1 (275/450) | N/A | 64.0  (48/75) | 56.0  (42/75) | 45.0  (9/20) | 42.7  (32/75) | 69.3  (52/75) | 94.2  (49/52) | 68.8  (11/16) | 51.6  (32/62) |
| **AAV5 %**  **(+/total)** | 34.8 (188/540) | 26.9  (7/26) | 37.2  (32/86) | 28.1  (25/89) | 40.0  (8/20) | 29.8  (25/84) | 46.2  (42/91) | 51.8  (29/56) | 5.9  (1/17) | 26.8  (19/71) |
| Adolescents | 28.8  (19/66) | 0/3 | 18.2  (2/11) | 7.1  (1/14) | 0/0 | 50.0  (4/8) | 43.8  (7/16) | 25.0  (1/4) | 0/1 | 44.4  (4/9) |
| Adults | 35.7 (169/474) | 30.4  (7/23) | 40.0  (30/75) | 32.0  (24/75) | 40.0  (8/20) | 27.6  (21/76) | 46.7  (35/75) | 53.8  (28/52) | 6.3  (1/16) | 24.2  (15/62) |
| **AAV6 %**  **(+/total)** | 48.7 (250/513) | N/A | 54.7  (47/86) | 43.8  (39/89) | 40.0  (8/20) | 31.3  (26/83) | 56.0  (51/91) | 80.4  (45/56) | 41.2  (7/17) | 38.0  (27/71) |
| Adolescents | 27.0  (17/63) | N/A | 18.2  (2/11) | 7.1  (1/14) | 0/0 | 37.5  (3/8) | 31.3  (5/16) | 100  (4/4) | 0/1 | 22.2  (2/9) |
| Adults | 51.8  (233/450) | N/A | 60.0  (45/75) | 50.7  (38/75) | 40.0  (8/20) | 30.7  (23/75) | 61.3  (46/75) | 78.8  (41/52) | 43.8  (7/16) | 40.3  (25/62) |
| **AAV8 %**  **(+/total)** | 45.6 (234/513) | N/A | 45.3  (39/86) | 42.7  (38/89) | 40.0  (8/20) | 38.6  (32/83) | 53.8  (49/91) | 69.6  (39/56) | 41.2  (7/17) | 31.0  (22/71) |
| Adolescents | 36.5  (23/63) | N/A | 27.3  (3/11) | 28.6  (4/14) | 0/0 | 37.5  (3/8) | 31.3  (5/16) | 100  (4/4) | 100  (1/1) | 33.3  (3/9) |
| Adults | 46.9  (211/450) | N/A | 48.0  (36/75) | 45.3  (34/75) | 40.0  (8/20) | 38.7  (29/75) | 58.7  (44/75) | 67.3  (35/52) | 37.5  (6/16) | 30.6  (19/62) |
| **AAVrh10 % (+/total)** | 46.0 (236/513) | N/A | 52.3  (45/86) | 37.1  (33/89) | 50.0  (10/20) | 30.1  (25/83) | 52.7  (48/91) | 73.2  (41/56) | 52.9  (9/17) | 35.2  (25/71) |
| Adolescents | 27.0  (17/63) | N/A | 18.2  (2/11) | 7.1  (1/14) | 0/0 | 50.0  (4/8) | 18.8  (3/16) | 100  (4/4) | 0/1 | 33.3  (3/9) |
| Adults | 48.7  (219/450) | N/A | 57.3  (43/75) | 42.7  (32/75) | 50.0  (10/20) | 28.0  (21/75) | 60.0  (45/75) | 71.2  (37/52) | 56.3  (9/16) | 35.5  (22/62) |

Samples from Brazil were only tested using validated assays, not RUO assay. Adolescents are <18 years of age, adults are ≥18 years. Numbers in parentheses are numbers of positive samples and total samples.

+, positive; AAV, adeno‐associated virus; N/A, not available; RUO, research-use-only.
