# Supplementary material for: Global Seroprevalence of Pre-existing Immunity Against AAV5 and Other AAV Serotypes in People with Hemophilia A
Source: Hum Gene Ther. 2022 Apr 19;33(7-8):432–41. doi: 10.1089/hum.2021.287 (PMC9063149; doi:10.1089/hum.2021.287)
Supplement: Supplemental data [file Suppl_FigS2.docx]

**Supplementary Figure 2.** Mean titer for all AAV serotypes globally and by country


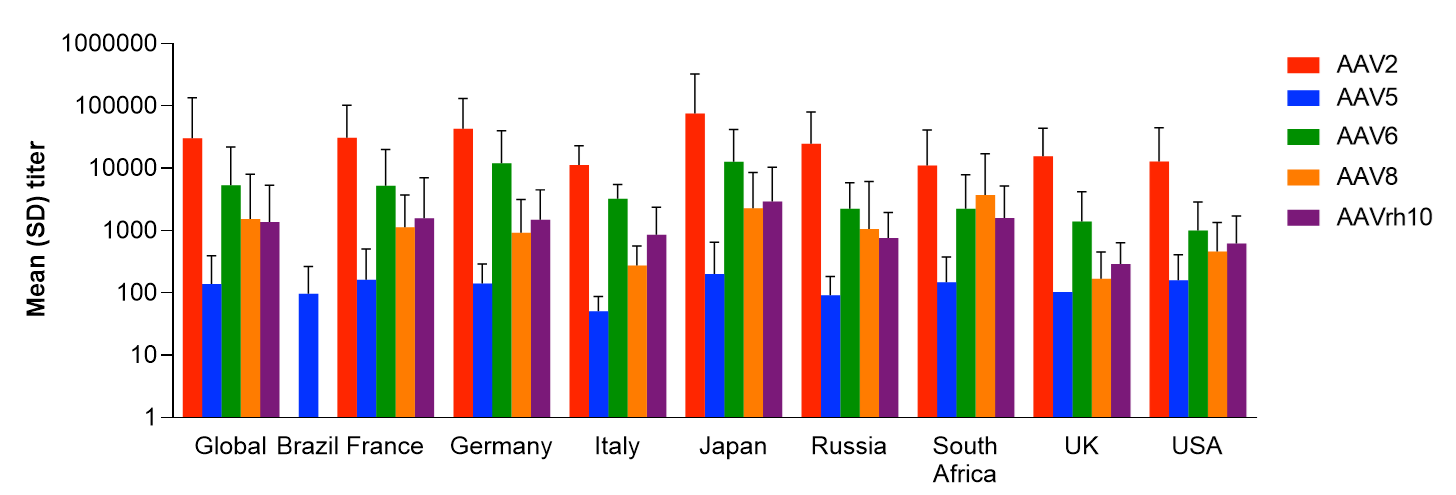


The detection threshold for the titer assay was 20, corresponding to the minimum required dilution. Samples from Brazil were only tested using the AAV5 assay, not RUO assays. Titer was only evaluated in seropositive participants. Note that titers cannot be directly compared across serotypes, as assays have varying sensitivity.

AAV, adeno-associated virus; RUO, research-use-only; SD, standard deviation.
