# Supplementary material for: Global Seroprevalence of Pre-existing Immunity Against AAV5 and Other AAV Serotypes in People with Hemophilia A
Source: Hum Gene Ther. 2022 Apr 19;33(7-8):432–41. doi: 10.1089/hum.2021.287 (PMC9063149; doi:10.1089/hum.2021.287)
Supplement: Supplemental data [file Suppl_TableS3.docx]

**Supplementary Table 3.** AAV serotype Day 1 positivity frequency by history of HIV and hepatitis B and C infection

| **AAV Serotype** | **HIV+** | **HIV−** | **Hepatitis B+** | **Hepatitis B−** | **Hepatitis C+** | **Hepatitis C−** |
| --- | --- | --- | --- | --- | --- | --- |
| **AAV2 %**  **(+/total)** | 59.1  (13/22) | 58.5  (287/491) | 56.0  (56/100) | 59.5  (235/395) | 61.5  (168/273) | 54.9  (128/233) |
| **AAV5 %**  **(+/total)** | 30.4  (7/23) | 35.0  (181/517) | 35.0  (36/103) | 35.1  (147/419) | 38.8  (109/281) | 31.0  (78/252) |
| **AAV6 %**  **(+/total)** | 36.4  (8/22) | 49.3  (242/491) | 46.0  (46/100) | 50.4  (199/395) | 51.3  (140/273) | 46.4  (108/233) |
| **AAV8 %**  **(+/total)** | 50.0  (11/22) | 45.4  (223/491) | 46.0  (46/100) | 46.1  (182/395) | 48.4  (132/273) | 42.5  (99/233) |
| **AAVrh10 % (+/total)** | 36.4  (8/22) | 46.4  (228/491) | 44.0  (44/100) | 47.1  (186/395) | 48.7  (133/273) | 42.9  (100/233) |

Data are for the global population on Day 1. Numbers in parentheses are numbers of positive samples and total samples.

AAV, adeno-associated virus; HIV, human immunodeficiency virus.
